# Service and Policy implication of substance use disorders among adolescents in juvenile correctional facilities in Lagos, Nigeria

**DOI:** 10.1017/gmh.2016.25

**Published:** 2016-11-07

**Authors:** O. Atilola, B. Ola, G. Abiri

**Affiliations:** 1 Department of Behavioural Medicine, Lagos State University College of Medicine, Ikeja, Lagos, Nigeria; 2 Department of Psychiatry, Lagos State University Teaching Hospital Ikeja, Lagos, Nigeria; 3 Child and Adolescent Unit, Federal Neuro-psychiatric Hospital Yaba, Lagos, Nigeria

**Keywords:** Adolescent, child mental health services, correctional services, juvenile justice, Nigeria, substance use disorder

## Abstract

**Background.:**

Lack of relevant data has continued to militate against the development of policy and practice toward identification and treatment of alcohol/substance abuse among adolescents coming in contact with the juvenile justice system in Nigeria. This study aims to provide such data, including its policy/practice implications.

**Methods.:**

One hundred and seventy eight (178) adolescents, who are representative of adolescents within the youth correctional services of Lagos jurisdiction, were interviewed using the alcohol and substance abuse section of the Kiddies’ Schedule for Affective Disorders and Schizophrenia.

**Results.:**

The lifetime prevalence rate of abuse of/dependence on any of alcohol or other substances was 22.5% (alcohol, 12.3%; illicit substance, 17.9%). Males were overrepresented among those with any substance use disorder, with gendered prevalence rate as high as 35%. Having had a lived-experience of being a street-child was the single most significant independent factor (Odds ratio (OR), 8.4; *p* = 0.007) associated with lifetime alcohol substance use disorder.

**Conclusions.:**

Substance use disorder is highly prevalent among adolescents within the juvenile justice systems in Lagos Nigeria. There is need for deliberate incorporation of alcohol and substance abuse screening and intervention as part of individual care plan in youth correctional facilities in Nigeria. Practical steps toward achieving this were drawn from local reality and international best practices.

## Introduction

Official reports from the past couple of years show that the number of adolescents coming in contact with the youth correctional systems in Nigeria is on the rise (Ogundipe, 2011). This is perhaps a direct consequence of worsening social and economic challenges in the country. Economic and social challenges engender an increased risk of juvenile delinquency on one hand, and also increase the risks of adolescent substance abuse on the other (Siegel & Welsh, 2008). It has also been suggested that the higher risk-taking behaviors, which may naturally come with adolescent substance abuse, increase the chances of offending and consequent juvenile justice contact (Siegel & Welsh, 2008). In other words, the risk factors for juvenile delinquency, youth offending, and adolescent substance abuse appears inter-related and cross-cutting. As a matter of fact, substance abuse among young persons has been viewed as a variant of juvenile delinquency *sue generis* and an integral part of the youth offending syndrome (Neff & Waite, 2007; Ogunwale *et al*. 2011). It is therefore not surprising that research from different parts of the world have documented high prevalence rates of co-morbid substance abuse among youth in correctional institutions around the world (Teplin *et al.* 2002; Wasserman *et al.* 2002; Prichard & Payne, 2005; Ogunwale *et al.* 2011).

When left unaddressed, substance abuse among young offenders reduces the chance of successful reformation and rehabilitation, and increases the risk of criminal recidivism (Cottle *et al.* 2001). Therefore, incorporating substance abuse screening and treatment into correctional services is very critical. In spite of the preponderance of evidence to justify a need for such, a significant proportion of youth correctional centers around the world lacked integrated substance abuse screening and treatment services (Snyder & Sickmund, 2006; Young *et al.* 2007). The situation is even worse in Nigeria as substance abuse is yet to be identified or prioritized as an issue of concern among adolescents within correctional institutions, and there is yet to be any clear-cut policy on drug abuse assessment and treatment in such institutions in the country (Ogunwale *et al.* 2011).

There is need for deliberate formulation of policies geared toward pre-empting, preventing, and addressing substance abuse among adolescents coming (or at-risk of coming) in contact with the youth correctional systems in Nigeria. A required critical step toward achieving this is availability of data on the nature and pattern of substance use problems among adolescents in correctional institutions in the country. Currently available epidemiological data on substance abuse among residents of youth correctional facilities in Nigeria is still limited in number and methodological robustness. For instance, only a single published study has deliberately focused on substance abuse among youth in correctional facilities in Nigeria (Ogunwale *et al.* 2011). This particular study was however male gender-biased; used a small sample size, and focused on more serious offenders in a special facility (Ogunwale *et al.* 2011). More importantly, the study focused only on self-reported use without establishing problematic pattern of use such as abuse or dependence. The only other available data on substance use/abuse among youth in correctional facilities in Nigeria is what can be gleaned from the equally limited number of studies that have assessed for general psychopathology among institutionalized children and adolescents (Ajiboye *et al.* 2009; Atilola, 2012*a*). Stimulating policy review will require strong and loud evidence, especially in a country such as Nigeria where child mental health policy issues are often relegated (Atilola *et al.* 2015).

The present study therefore examines the lifetime prevalence rate of problematic pattern of use (abuse and dependence) of alcohol and other substances among adolescents in youth correctional facilities in Lagos Nigeria. Unlike the previous studies, the present study used large and representative sample of adolescent boys and girls and included a discussion on the policy implications of the results for the management of substance use disorders among youth in correctional facilities in Nigeria.

## Methods

This research is part of a larger research seeking to provide rationale and framework for incorporating mental health screening and interventions into juvenile justice services in Lagos, Nigeria.

### Settings

The study was conducted in all the five youth correctional facilities being operated within the Lagos city. These facilities include Special Correctional Centre for Girls Idi-Araba; Special Correctional Centre for Boys Oregun, Correctional Centre for Senior Boys Isheri, Correctional Centre for Junior Boys Birrel, Sabo-Yaba, and the Correctional Centre for Senior Girls Idi Araba. When an adolescent runs in conflict with the law or is adjudged to be in danger within the jurisdiction of Lagos, he/she is brought to the juvenile courts by the Lagos juvenile police. Depending on the facts of the case, the juvenile court may release such adolescent or issue a committal order into a correctional institution.

### Sampling

Proportionate sampling was used in all the five youth correctional facilities. Two-thirds of all the adolescents in the institution as at the time of study were sampled. This was done by first stratifying the adolescents in each institution based on the three categories of adolescents in each. These categories normally include: (1) juvenile offenders, (2) adolescents adjudged to be beyond parental control, and (3) vulnerable adolescents in need of care and/or protection of the state. Two-thirds of the total number of adolescents under each category was selected, giving rise to a proportionate (based on category) two-thirds of adolescents in the institution. Simple random sampling in which two of every three adolescents on the institutional register (which has been stratified into the three categories and in no particular order) was used to select participants in each institution.

### Instruments and procedures

In this particular report, basic socio-demographic data on age, gender, and reason for admission in the correctional facility were obtained using a pre-designed proforma. A retrospective diagnosis of lifetime alcohol and substance abuse/dependence was made using the substance abuse section of the Kiddies’ Schedule for Affective Disorders and Schizophrenia (K-SADS) (Kaufman *et al.* 1997). The K-SADS is a semi-structured diagnostic interview designed to assess psychopathology in children and adolescents in accordance with the fourth edition of the Diagnostic and Statistical Manual (DSM-IV) criteria (American Psychiatric Association, 2000). It can be used by trained professionals to assess for the presence or otherwise of DSM-IV psychiatric disorders including substance use disorders. All assessments were conducted by psychiatrists who are trained and experienced in the use of K-SADS. The interviews were face-to-face, but in private. Since adolescents were never allowed passes or home visits until they are finally discharged from the institutions, substance-use enquiry was limited to the experience of the adolescents prior to their admission into the institutions.

### Statistics

All data were entered, cleaned, and analyzed using SPSS (IBM, Chicago, IL, USA) version 16. The unadjusted relationship between substance use disorder and categorical variables such as gender, admission category, and educational history were analyzed using chi square tests. Relationship between substance use disorder and continuous variables such as age and length of lived-experience of street life were analyzed using Student's *t* test. Values of *p* < 0.05 was considered statistically significant. Logistic regression was used to determine independent associations with substance abuse/dependence.

## Results

A total of 185 residents of correctional facilities across Lagos were sampled, but seven were excluded from the study due to inability to comprehend the interview for reasons ranging from severe mental disorder, intellectual disabilities, or inability to understand any of English or Yoruba languages. In all, 178 residents completed the study. There were more males (*n* = 110; 61.8%) than females. The overall mean age was 15.19 ± 1.98 years. The participants had stayed in the institutions for a period that ranged from as recent as 1 week to as long as 120 months. The median length of stay was 12 months. Prior to being admitted into these centers, the participants had lived by themselves as a child or had lived in the streets for a median period of 2 months (range from 1 week to 72 months).

Majority (73.6%) of the participants were remanded in the institutions for care and protection by the state, mostly as run-away kids, street-hawkers, or otherwise found lost or wandering in the city. The rest of the participants were remanded as young offenders (mostly for theft) or those declared as beyond parental control (see Table 1). Furthermore, when compared with the girls, boys were significantly older (*t* = −2.12; *p* = 0.035); more likely to be in the ‘offender’ category, and more likely to have pre-existing school-related problems (such as truancy, school refusal) before being remanded (see Table 1). As shown in Table 1, the boys were also more likely to have lived by themselves/have lived-experience of street-life (*χ*^2^ = 4.8; *p* = 0.028) and for a significantly longer period (*p* < 0.05) compared with girls. Table 1 shows other details of socio-demographic characteristics of participants.

**Table 1.** Socio-demographic characteristics of respondents (*n* = 178; 100%)

Variable	Total (*n* = 178; 100%)	Gender distribution	Statistic	*p*
	Male *v.* female			
Age in years (mean)	15.19 ± 1.98	15.43 ± 1.94 *v.* 14.79 ± 1.98	−2.12^a^	**0**.**035**
Reason for admission into the correctional facility				
Young offender	34 (19.1)	30 (88.2) *v.* 4 (11.4)		
Accused of or remanded for theft/larceny/burglary	*19 (10.7)*			
Accused of or remanded for perpetrating physical assault/involvement in gang activities	*6 (3.4)*			
Accused of or remanded for murder/manslaughter	*5 (2.8)*			
Remanded for trespass of an aircraft (stowaway) or other private properties	*2 (1.1)*			
Accused of or remanded for being a perpetrator of sexual assault/rape	*1 (0.6)*			
Beyond parental control	13 (7.3)	8 (61.5) *v.* 5 (38.5)		
Brought to the juvenile police by parents/guardian due to recurrent violation of rules				
Care and protection of the State	131 (73.6)	72 (55.0) *v.* 59 (45.0)	12.6^b^	**0.020**
Run-away/street-hawking kid apprehended by police task-force during a street raid	*74 (41.6)*			
Handed over to juvenile police by concerned citizens having been found lost or wandering	*34 (19.1)*			
Victim of sexual abuse	*10 (5.6)*			
Victim of recurrent or single (major) physical maltreatment	*4 (2.2)*			
Known orphan or abandoned child	*4 (2.2)*			
Victim of forced child-labor	*3 (1.7)*			
Remanded for under-age pregnancy	*1 (0.6)*			
Institutionalization/recidivism				
Length of continuous stay within the institution (months)^c^				
Median length (12 months) and below		67 (71.3) *v.* 27 (28.7)		
Above the median length		43 (51.2) *v.* 41 (48.8)	7.58^d^	**0.006**
Previous admissions into the institution (recidivism)				
Yes	17 (9.6)	12 (70.6) *v.* 5 (29.4)	2.19^d^	0.334
Educational history				
School status as at the point of admission into institution				
In school^e^	63 (35.4)	33 (52.4) *v.* 30 (47.6)		
Dropped out of school	89 (50.0)	65 (73.0) *v.* 24 (27.0)		
Never ever in school	26 (14.6)	12 (46.2) *v.* 14 (53.8)	9.99^b^	**0.007**
Self-reported preexisting school-related problems before admission into institution^f^				
Repeated a grade	47 (26.4)	34 (72.3) *v.* 13 (27.7)	18.4^d^	**<0.001**
School-refusal	28 (16.7)	22 (78.6) *v.* 6 (21.4)	9.3^d^	**0.009**
Truancy	33 (18.5)	27 (81.8) *v.* 6 (18.2)	10.7^d^	**0.005**
Pre-admission skill acquisition				
Ever registered to learnt a trade	88 (49.4)	71 (80.7) *v.* 17 (19.3)	26.2^d^	**<0.001**
Completed the training to the point of certification	9 (5.1)			
Experience of street-life/living alone				
Ever had to live on the streets or by self^g^	84 (47.2)	59 (70.2) *v.* 25 (29.8)	4.8^d^	**0.028**
Length of staying on the streets or by self (months)				
Median length (2 months) and below		20 (45.5) *v.* 24 (54.5)		
Above the median length		37 (97.4) *v.* 1 (2.6)		**<0.001**^h^

a Student's *t* test.

b Likelihood ratio.

c Some were transferred from social-welfare homes such as orphanages.

d Pearsons *χ*^2^.

e Must have attended school up till 2 weeks before being admitted in the institution.

f If ever in school.

g Before the age of 18 years.

h Fisher's exact test.

The lifetime prevalence rate of abuse of/dependence on any of alcohol or other substances was 22.5% (*n* = 40). For alcohol abuse/dependence alone, the lifetime prevalence was 12.3% (*n* = 22), while that of substance abuse/dependence was 17.9% (*n* = 32). Males were overrepresented (about 95%) among those with any lifetime alcohol/substance use disorders, with a gendered prevalence rate of 34.9%. Cannabis was overwhelmingly (88%) the most common substance of abuse/dependence. Others were tobacco, inhalants/solvents, cocaine, and codeine. Using more than planned, tolerance, and frequent reinstatement after taking decision to quit were the most common problematic pattern of use of either alcohol or other substances (Table 2).

**Table 2.** Frequency of problematic pattern of use of alcohol and/or other substance among residents of youth correctional facilities in Lagos

Problematic pattern	Total (*n*; %)	Alcohol (*n*; %)	Other substances (*n*; %)
Using more than planned	20 (11.2)	16 (9.0)	14 (7.8)
Continued use despite negative physical consequences	14 (7.9)	8 (4.5)	6 (3.4)
Continued use despite engendering of dangerous behaviors	11 (6.2)	6 (3.4)	5 (2.8)
Continued use despite negative psychological consequences	4 (2.2)	2 (1.1)	2 (1.1)
Continued use despite negative affectation of occupational functioning	5 (2.8)	4 (2.2)	1 (0.6)
Continued use despite other negative social consequences	6 (3.4)	4 (2.2)	2 (1.1)
Continued use despite legal consequences	5 (5.8)	1 (0.6)	4 (2.2)
Continued use despite getting intoxicated at major role obligations	4 (2.2)	2 (1.1)	2 (1.1)
Giving up important activities for substance use	2 (1.1)	1 (0.6)	1 (0.6)
Tolerance	21 (11.8)	10 (5.6)	11 (6.1)
Frequent reinstatement of substance after initial quits	32 (20.0)	16 (8.9)	16 (8.1)
Experience of withdrawal symptoms	12 (6.7)	4 (2.2)	8 (4.5)
Relief of withdrawal symptoms with use	10 (5.6)	4 (2.2)	6 (3.4)

At bivariate analysis (Table 3), a significantly higher proportion of participants who were remanded under the ‘young offender’ category met criteria for lifetime substance use disorder compared with those under the care and protection and beyond-parental-control category (*χ*^2^ = 11.2; df = 2; *p* = 0.004). Although other socio-demographic factors such as age, gender, previous history of truancy, and history of living by self or on the streets were all significantly (*p* < 0.05) associated with alcohol/substance abuse/dependence at bivariate analyses (Table 3); having lived by self in shelter or in the streets was the sole factor independently associated with substance abuse/dependence after multivariate analysis (Table 4).

**Table 3.** Correlates of problematic use (abuse/dependence) of alcohol and/or other substance among residents of youth correctional facilities in Lagos

Variable	Problematic use (abuse or dependence)		*χ*^2^	*p*
	Yes (*n* = 40)	No (*n* = 138)		
Mean age	15.77 ± 1.86	15.02 ± 1.99	−2.137^a^	**0.034**
Gender				
Male (*n* = 110)	38 (34.9)	72 (65.1)		<0.001^**b**^
Female (*n* = 68)	2 (2.9)	66 (97.1)		
Reason for admission into the correctional facility				
Young offender(*n* = 34)	15 (44.1)	19 (55.9)		**0.004**
Beyond parental control (*n* = 13)	1 (7.7)	12 (82.3)		
Care of State (131)	24 (18.3)	107 (81.7)	11.2^c^	
School status as at the point of admission into institution				
In school^d^ (*n* = 63)	10 (15.1)	53 (84.1)		0.183
Dropped out of school (*n* = 89)	25 (28.1)	64 (79.1)		
Never ever in school (26)	5 (19.2)	21 (80.8)	3.39^c^	
Self-reported preexisting school-related problems before admission into institution^e^				
Repeated a grade (*n* = 47)				
Yes	13 (27.7)	34 (72.3)	0.533	0.465
No	27 (22.3)	94 (77.7)		
School-refusal (*n* = 28)				
Yes	10 (35.7)	18 (64.3)		
No	26 (20.3)	102 (79.7)	3.07	0.081
Truancy (*n* = 33)				
Yes	15 (45.5)	18 (54.5)		
No	21 (18.1)	95 (81.9)	10.49	**0.001**
Pre-admission skill acquisition				
Ever registered to learnt a trade (*n* = 88)				
Yes	30 (34.1)	58 (65.9)		
No	10 (11.1)	80 (88.9)	13.48	**<0.001**
Experience of street-life/living alone				
Ever had to live on the streets or by self^f^				
Yes	23 (27.4)	61 (72.6)		
No	17 (18.1)	77 (81.9)	2.20	0.096
Length of staying on the streets or by self (months)				
Median length (2 months) and below	2 (4.5)	42 (95.5)		
Above the median length	20 (52.6)	18 (47.4)		**<0.001**

a Student's *t* test.

b Fisher's exact statistic.

c Likelihood ratio.

d Must have attended school up till 2 weeks before being admitted in the institution.

e If ever in school.

f Before the age of 18 years.

**Table 4.** Socio-demographic factors that are independently associated with problematic (abuse/dependent) use of alcohol and/or other substance among residents of youth correctional facilities in Lagos

Characteristics	Odds ratio	95% confidence interval	*p*
Length of staying on the streets or by self (months)			
Median length (2 months) and below	1		0.007
Above the median length	8.44	2.08–26.27	

Other variables in equation: age, gender, reason for admission, pre-admission skill acquisition, and experience of street-life/living alone.

## Discussion

The present study found a lifetime prevalence rate of 22.5% for any substance use disorder (abuse of or dependence on alcohol and/or other substances) among the adolescents in the youth correctional services in Lagos. This finding is similar to the findings in other Nigerian studies, which used similar methodology. For instance, Atilola (2012*a*) recorded a 28% prevalence rate of any substance use disorder, while Ajiboye *et al.* (2009) recorded a prevalence rate of 26.4% among adolescents in a Remand home in Ibadan and a Borstal home in Ilorin, respectively. However, the prevalence rates of substance use disorder reported in the present and earlier studies as cited above are a bit higher than the 21% prevalence rate reported among school-going adolescents in Nigeria (Atilola *et al.* 2013). Adolescents drawn from juvenile-justice populations are known to have higher risk factors for substance abuse compared with non-delinquent adolescent populations (Siegel & Welsh, 2008).

In terms of specific substances, at lifetime prevalence rates of 12.3% and 17.9%, respectively, alcohol and cannabis stood out distinctly as the most commonly abused substances among the participants in the present study. Other studies which have examined substance use disorders among similar population in different parts of the world including Nigeria had found alcohol and cannabis as among the most widely abused substances (e.g. Teplin *et al.* 2002; Abram *et al.* 2003; McClelland *et al.* 2004; Ajiboye *et al.* 2009). This finding is in keeping with the earlier report that at the point of debut of substance use (which often happens during adolescence) alcohol and cannabis are the most commonly used substances among adults in Nigeria (Gureje *et al.* 2007). Aside this, alcohol is also the most easily accessible and socially acceptable licit substance in many other parts of the world (United Nations Office on Drugs and Crime, 2010), while cannabis has somehow acquired notoriety as the most widely used illicit substance globally (United Nations Office on Drugs and Crime, 2010).

Beyond occasional lifetime use, an emerging conclusion from currently available data (Atilola, 2012*a*; Ajiboye *et al.* 2009; Ogunwale *et al.* 2011) including the present study is that alcohol and cannabis use disorders are common lifetime psychopathologies among adolescents who come in contact with youth correctional institutions in Nigeria. This conclusion is not at all surprising, as there had been robust research evidence for a strong association between substance abuse and juvenile delinquency (Young *et al.* 2007; Tripodi & Bender, 2011). Furthermore, several studies conducted in other parts of the world have unequivocally established high prevalence rates of lifetime substance use disorders among adolescents who interface with the juvenile justice system (Teplin *et al.* 2002; Abram *et al.* 2003; McClelland *et al.* 2004; Prichard & Payne, 2005).

Strikingly though, the prevalence rate of 22.5% lifetime abuse of and/or dependence on any substance recorded in the present study is far lower than the average of about 50% (range of 48%–52%), which has been recorded in other similar studies outside Nigeria (Teplin *et al.* 2002; Abram *et al.* 2003; McClelland *et al.* 2004). This may simply be as a result of recall bias, as unlike in other studies where data were obtained early in the course of incarceration, the respondents in the present study had stayed in the institution for a median period of about 12 months and may have forgotten some details about their substance use history. In the alternative, a closer look at the demographics of the participants in the present study also points to other possible explanations for the disparities. Over 70% of the participants in the present study have committed minor or status offences or are in fact victims of abuse and neglect. Unlike in most part of the developed world where the juvenile justice system has fully evolved and where residents of juvenile justice institutions are mostly serious offenders; juvenile justice institutions in Nigeria still continue to be populated mostly by minor or status offenders, and at times those admitted for protective custody (Atilola *et al*. 2013). Socio-ecological links between juvenile crime and substance abuse dictates that the tendency to engage in substance abuse tends to have direct relationship with the severity of crime (Young *et al.* 2007; Tripodi & Bender, 2011). Hence, a higher prevalence of substance use disorder is expected among populations of serious offenders compared with the case in the present study where youth correctional populations are mostly minor/status offenders. This assertion is buttressed by the observation that more serious offenders in the present study had a statistically significant higher prevalence of lifetime substance use disorder compared with the less serious offenders and those on protective custody.

An overwhelming majority of the participants in the present study who had lifetime substance use disorders were males. This finding is also different from the reports from other parts of the world where only a slight difference has been found in the prevalence rates of substance use disorders among male and female juvenile detainees (Teplin *et al.* 2002; Abram *et al.* 2003; McClelland *et al.* 2004). Again, a closer look at the present data shows that the males in the present study differs statistically from the females in such a way that may explain the sharp difference in the prevalence rates of substance use disorders among the two groups. In-depth analyses have shown that gender difference in the prevalence rates of alcohol and substance abuse among incarcerated youth, where exists, is more a reflection of the differential seriousness of offense among the males and females (Neff & Waite, 2007). Unlike the situation in other parts of the world where incarcerated youth are often serious offenders irrespective of gender, significantly more boys in the present study were serious offenders and recidivists compared with girls. In addition, other pre-delinquent social problems, which may favor exposure to substance abuse such as living alone as a child, lived-experience of street life, truancy and other school-related problems were all significantly more common among boys than girls in the present study.

### Policy implications for drug abuse recognition and treatment in juvenile correctional settings in Nigeria

Having discussed our findings in context of extant literature, we also need to highlight how these findings could aid the development of mental-health policy for this juvenile population. The present study has emphasized further that substance abuse and dependence are among the pre-contact social/mental health problems among adolescents that are brought before the juvenile justice system in Nigeria. This observation has implications for pre-emptive juvenile justice administration in Nigeria. It is known that substance use disorder can accelerate the decent of an otherwise delinquent youth into more serious offending and subsequent juvenile justice contact (Siegel & Welsh, 2008). Therefore, a key pre-emptive step is to attempt to break the cycle of substance use disorder and subsequent juvenile justice contact among vulnerable youth. This is best done by understanding the dynamics of the factors that might be promoting substance abuse among at-risk youth before eventual juvenile justice contact. Differential association/social learning theorists have suggested that the more the frequency and endurance of the exposure of a young person to settings where substance abuse is seen as acceptable, the more likely that they will engage in it (Neff & Waite, 2007). In other words, the more enduring the exposure to social contexts where substance abuse constitutes a positive behavior, the more likely for a young person to adopt such behaviors. The single most important social-context, which was independently associated with pre-contact substance use disorder among residents of correctional facilities in the present study, is the length of their stay on the streets before juvenile justice contact. At least one other similar study conducted in another youth correctional institution in Nigeria has made same observation (Atilola, 2012*b*).

The inner streets of most Nigerian cities are common settings where young persons may encounter other peers with permissive attitude toward substance use and delinquent behaviors (Morakinyo & Odejide, 2003; Olley, 2006). Therefore, reducing the influx of youth to the streets, and reducing the length of their stay on the street before intervention, can be a long-term strategy of reducing youth substance abuse and subsequent juvenile justice contact in Nigeria. Poverty and social inequalities and the search for escape from life of deprivation, hardship, and abuse is at the heart of influx of children and youth into the streets in Nigeria (Ebigbo, 2003; Omiyinka, 2009). Child-sensitive social security systems such as support for vulnerable families can be a long-term solution. More specifically, a school-reintegration system in which ‘arrested’ street-children are placed in schools with conditional support can be a medium-term approach to psychoactive substance and youth crime. The conditionality in this case can be in the form of compulsory referral to substance abuse treatment (if indicated) and/or cash-transfers predicated on compulsory school attendance or vocational training. This approach can also be a form of diversion program, that is, strategies embedded within the youth justice system to divert offending youth into other judicial programs aimed at preventing them from formal court action and incarceration (Muncie, 1999). Such diversion programs have been recommended as modality of choice for juvenile justice populations such as those seen in Nigeria, which are predominantly status- and minor-offenders (Atilola *et al*. 2013).

Furthermore, diversion programs have the additional advantage of addressing, in part, the operative practice of having to lump together adolescents who are serious offenders, minor/status offenders, as well as victims of abuse/neglect together in same incarcerating facility as observed in the present study. This situation is challenging because in the absence of a program to address substance use disorder, as is the case in most youth correctional services in Nigeria (Ogunwale *et al*. 2011), there is a risk that adolescents with little or no prior exposure to substance use may get exposed to substance use behaviors. More importantly, diversion programs will ensure that non-offenders and minor/status offenders are diverted into community programs, while the youth correctional system will have more time, space, and resources to focus on more serious offenders (who also have a higher prevalence of substance use disorders as shown in the present study). The importance of and the modalities for separating victims from offenders within the juvenile justice systems in Nigeria has been discussed elsewhere (Atilola *et al*. 2013).

Beyond efforts aimed at diverting youth away from the streets and incarcerating facilities, present realities suggest, in line with Ogunwale *et al*. (2011) that the continued lack of a clear-cut policy on drug abuse assessment and treatment within the juvenile justice institutions in Nigeria needs to be urgently addressed. Reports from other parts of the world shows that the presence of a policy on substance use screening/treatment in youth correctional institutions ensured successful rehabilitation and reintegration of wards compared with similar facilities who lacks such policy (CASA, 2004). In fact, there is illustrative evidence in Nigeria, which shows that when re-arrested and brought back into the juvenile institution, adolescents who reported substance use in their first contact with the juvenile institutions had continued to use the same substance or more at the point of second arrest (Ogunwale *et al*. 2011). In other words, substance abuse remains a co-occurring and co-morbid problem among adolescents even after contact with the juvenile justice system. Therefore it is important to start evolving evidence-based policy that highlights strategies for substance abuse identification and assessment within the Nigerian juvenile justice systems through a blend of indigenous knowledge and available global best practices.

Based on the findings of the present study, a key step toward substance abuse treatment for adolescents coming in contact with correctional services in Lagos and other parts of Nigeria will be to incorporate an entry-point compulsory evaluation for substance abuse. Although the legal framework for juvenile justice administration in Nigeria is still generally incoherent and inchoate at present (Atilola *et al*. 2013), there is still a provision for pre-intake medical examination in some of the extant legal frameworks such as the Section 4, Subsection 14 of Borstal Institution and Remand Centre Act (Federal Ministry of Justice, 2004). This legal framework can be easily stretched to include full mental health and substance abuse screening. This can be in the form of a self-report assessment instruments with which adolescents can be screened at the point of entry. The ideal assessment instrument for such purposes should be brief, easy to administer by lay-persons, and have cut-offs for triaging substance abuse problems. Such instruments are available, and include the CRAFFT screening for Adolescent Substance Use Disorder (Knight *et al*. 2002), just to mention but one. Such instrument can then be tested in the juvenile justice population of Nigeria for psychometric properties (i.e. for reliability and validity). The fidelity of such adopted instrument can be further guaranteed through urine testing for alcohol and other substances.

Identification of adolescents with substance use disorders will facilitate treatment, including any other co-morbid mental health problems. Examples of behavioral treatments, which have shown efficacy in Western World are classical individualized cognitive behavioral therapy (CBT) approach (Turner, 1993; Kaminer *et al.* 2002), family-based therapies such as multidimensional family therapy (MDFT) or multisystemic family therapy (MST) (Liddle *et al*. 1993). In the setting of present study, individual therapy is likely to be more pragmatic than family-based interventions because being a juvenile justice setting, parents and families may not be readily available. In addition, a large proportion of adolescents in the present study have been disconnected with their parents and living on the streets for a significant length of time before coming in contact with the juvenile justice system. Nonetheless, the youth correctional system in Lagos has a family integration unit, which interphases with families of residents for the purpose of reintegration of street-exposed and runaway adolescents who comes in contact with the youth correctional system. This system could be explored for both family re-integration and the provision of family-based interventions substance abuse interventions. Family interventions, such as MDFT and MST, have been tested in high-intensity settings such as adolescent residential programs and have been shown to facilitate the reintegration of substance abusing juvenile detainees into the community (McHugh *et al.* 2010).

In summary, we align with other authorities (American Society of Addiction Medicine, 2001; McClelland *et al.* 2004) that comprehensive management of problematic substance use can be blended into juvenile justice system in several culturally competent ways and more importantly that coordination and collaboration between juvenile justice professionals, drug abuse treatment providers and other social service agencies are essential to facilitate this. For instance, while screening and assessment can be done upon arrest or referral, treatment can be initiated while waiting for trial. Moreover, treatment during incarceration can be followed by community based treatment after release (McClelland *et al.* 2004). In another way, access can be granted to treatment programs outside of correctional centers instead of incarceration (McClelland *et al.* 2004).

For practical purposes, a system can be created within the youth correctional services in Nigeria whereby after identification, culturally adapted individualized CBT- or family-based intervention can be administered. Both CBT- and MDFT-based treatments can be manualized and can be administered effectively in a 60–90-min weekly sessions (Liddle *et al.* 2008). The focus of such therapies should include motivation to stop or harm reduction. By the way, either in individual CBT- or family-based therapies, harm reduction often involves self-monitoring as one of the key strategies. The present study have identified the key indicators of descent into harmful use among participants to include using more than planned, tolerance, and frequent reinstatement after taking decision to quit. These ‘markers’ can be embedded into discussions about self-monitoring where complete abstinence is not feasible. Clinical psychologists or social workers with psychology bias can be trained and supervised to provide such interventions. While the authors are currently not privy to the personnel profile in the study setting, personal experience of authors is that psychologists and social workers with some training in psychology are often part of the staff composition of youth correctional facilities in Nigeria. Since there is currently no evidence for the efficacy of either CBT- or MDFT-based substance abuse interventions among juvenile justice populations in Nigeria, it will be more useful if proposed interventions are embedded within implementation research for now.

The present study has a lot of strengths. It went beyond assessment for substance use to establish problematic pattern of use (abuse and dependence). The sample size is large, gender balanced, and representative of juvenile justice population of a defined region (Lagos) in Nigeria. The policy implications of the findings are also extensively discussed. However, the study has some key limitations, which may have introduced some biases. Firstly, we relied exclusively on self-reports and as such, there could have been report biases. Secondly, the respondents have stayed within the institutions for periods sometimes as long as several months and as such, recall bias may be unavoidable when responding to questions that exclusively referred to periods before admission into the institution. A research design in which interviews were conducted at the point of entry into the institution, and where self-reports are corroborated with actual drug testing would have eliminated these biases to some extent. Finally, when comparing the results of research among juvenile justice populations around the world with that of Nigeria, researchers should be mindful of the unique and peculiar nature of these populations in Nigeria at present (Atilola *et al*, 2014).
